# Cataract surgery in patients with corneal opacities

**DOI:** 10.1186/s12886-018-0765-7

**Published:** 2018-04-23

**Authors:** Yi-Ju Ho, Chi-Chin Sun, Hung-Chi Chen

**Affiliations:** 1 Department of Ophthalmology, Chang Gung Memorial Hospital, Linkou, Taiwan; 2 Department of Ophthalmology, Chang Gung Memorial Hospital, 6F, Mai-Jing Road, An-Leh District, Keelung, Taiwan, Republic of China; 3 Department of Chinese Medicine, College of Medicine, Chang Gung University, Taoyuan, Taiwan

**Keywords:** Corneal opacities, Cataract, Phacoemulsification, Anterior segment optical coherence tomography, Corneal densitometry

## Abstract

**Background:**

Investigating the efficacy and safety of phacoemulsification with intraocular lens (IOL) implantation in corneal opacities.

**Methods:**

This retrospective study was conducted in a tertiary medical center. Twenty-three eyes of 19 patients with cataracts and corneal opacities obscuring the pupillary center having received phacoemulsification with IOL insertion without any ancillary techniques were enrolled. The primary study outcome measures were uncorrected and best corrected visual acuity (BCVA), and complications. Backscatters of corneal scar lesions were evaluated by slit lamp-based haze grading, Scheimpflug Pentacam and anterior segment optical coherence tomography (ASOCT). Visual outcomes after cataract surgeries and improvement range were used to determine the safety and efficacy of cataract surgery for our patients.

**Results:**

All patients underwent uneventful capsulorhexis and phacoemulsification. The mean age was 72.22 ± 10.1 years, and the mean follow-up period was 18.57 ± 15.42 months. The mean BCVA significantly improved from 1.45 ± 0.65 preoperatively to 0.94 ± 0.55 logMAR postoperatively (*p* < 0.001), and the number of eyes with a BCVA of 20/100 or better increased from 4 to 14. Complications included corneal edema in two eyes and reactivation of the previous corneal pathology in five eyes. Four eyes did not achieve an improvement in visual acuity after surgery, which may have been due to co-existing ocular co-morbidities. Both Pentacam corneal densitometry and ASOCT demonstrated no significant correlations with final visual outcome. However, a statistically significant relationship between the severity of corneal opacity and improvement range in BCVA (*r* = − 0.782, *P* = 0.001) was found by our OCT grading method.

**Conclusions:**

Phacoemulsification and IOL implantation in selected cases of coexisting cataracts and corneal opacities is safe that can provide suboptimal but long-term vision when penetrating keratoplasty is not possible or at high-risk of graft failure. ASOCT is a simple tool to predict visual outcomes after cataract surgery in opacified corneas.

## Background

Phacoemulsification is a standard cataract surgery with excellent outcomes. However, it is not unusual to encounter patients with coexisting corneal opacification and visually debilitating cataracts [1]. Corneal opacity can impede visualization during cataract surgery. There are two surgical options under such circumstances. The first is to perform simultaneous penetrating keratoplasty (PKP), cataract extraction and intraocular lens (IOL) implantation, which provides a shorter visual rehabilitation period [2]. However, the disadvantages include risk of expulsive hemorrhage, inadequate cortical cleaning and inaccuracy in IOL power calculation [3, 4]. The second option is to postpone cataract surgery after PKP to achieve refractive accuracy [5]. The drawbacks include a delay in visual rehabilitation and the risk of endothelial loss [4, 6]. In addition, factors such as graft rejection in high-risk recipients, poor patient compliance, meticulous follow-up and a paucity of good-quality donor corneas mean that immediate keratoplasty is often not possible. Therefore, cataract surgery alone can provide timely visual rehabilitation.

To enhance visibility during cataract surgery in opacified corneas, techniques such as capsule staining or alternative methods have been applied [7, 8]. However, toxicity of the staining dye should be taken into consideration, especially in a currently compromised cornea. Furthermore, uncontrolled dye dispersion through zonules can obscure the capsular anatomy [9]. With regards to modified illumination methods, transcorneal oblique illumination and endoscope-assisted phacoemulsification have been utilized, however, they are time consuming and require skilled clinicians [5, 10].

Variable outcomes have been reported after cataract surgery in eyes with corneal opacities [11, 12], and corneal opacity severity has been one of the major prognostic factors. Optical coherence tomography (OCT) has been used to investigate the histopathology of corneal opacities [13], and the degree of opacity has been reported a useful parameter in preoperative planning [14]. Corneal haze could hinder the light transmission resulting backward and forward scattering. Light-backscattering analysis with OCT seems to be a reliable method of detecting backscatter [15].

However, routine analysis has not been applied to evaluate the efficacy of cataract surgery in opacified corneas [16].

Recently, studies assessing the corneal pathology has included double-pass instrument for intraocular forward scattering [17], Scheimpflug rotating camera for backward scattering and corneal higher-order aberrations. Kamiya et al. suggested no significant correlations between visual performance and backward scattering in band keratopathy, while intraocular forward scattering maybe the only effective objective assessment for corneal pathology regarding visual acuity [18]. Although the correlation between reduced visual acuity and Scheimpflug corneal densitometry is not significant but still provide a quantitative value for backscatter in previous normative cohort study [19]; but in another study, significant level of correlations between visual performance was found in assessing granular corneal dystrophy [17]. Regarding backward scatter, previous study had demonstrated optical coherence tomography can precisely detect depth and visualize different corneal dystrophy in augment of treatment planning [20, 21]. But no quantitative analysis of the corneal opacities with visual performance was performed yet.

Therefore, the primary goal of this study was to report the visual outcomes and complications in patients undergoing cataract surgery in eyes with coexisting corneal opacities. The second aim was to evaluate whether anterior segment OCT can be used as an alternative objective modality to predict visual prognosis. To our knowledge, this is the first report to evaluate and compare non-transparent cornea with densitometry and new OCT grading methods.

## Methods

Medical records of patients with cataracts and corneal opacities who underwent phacoemulsification and IOL implantation were reviewed. Inclusion criteria were a best-corrected visual acuity (BCVA) less than 20/40 and corneal opacification involving the visual axis and advanced cataracts. Only eyes with partially visible anterior capsules and pupillary margins were enrolled. The exclusion criteria were use of ancillary techniques or those who received simultaneous keratoplasty. No patients had previously ocular surgeries except for one who had PKP before cataract surgery.

### Preoperative evaluation

The demographic and perioperative data were recorded. Visual acuity was expressed as logMAR. The severity of cataracts was categorized based on the Lens Opacity Classification System III [6]. Fundus examinations and B-scan ultrasonography were performed. Specular microscopy and anterior segment OCT were used to analyze the function and structure of the corneas.

### Slit lamp-based haze grading

We scored corneal haze in accordance with modified VISX protocol as follow: 0 indicates clear cornea and no haze; 0.5 is barely detectable haze; 1 is mildly affecting visual performance; 2 is moderated haze; 3 is opaque area prevent refraction, anterior chamber visible; 4 indicates opacity hinder view of anterior chamber. The cornea haze was scored by two observers (YJH and CCS).

### Corneal opacification grading

**Fig. 1:**
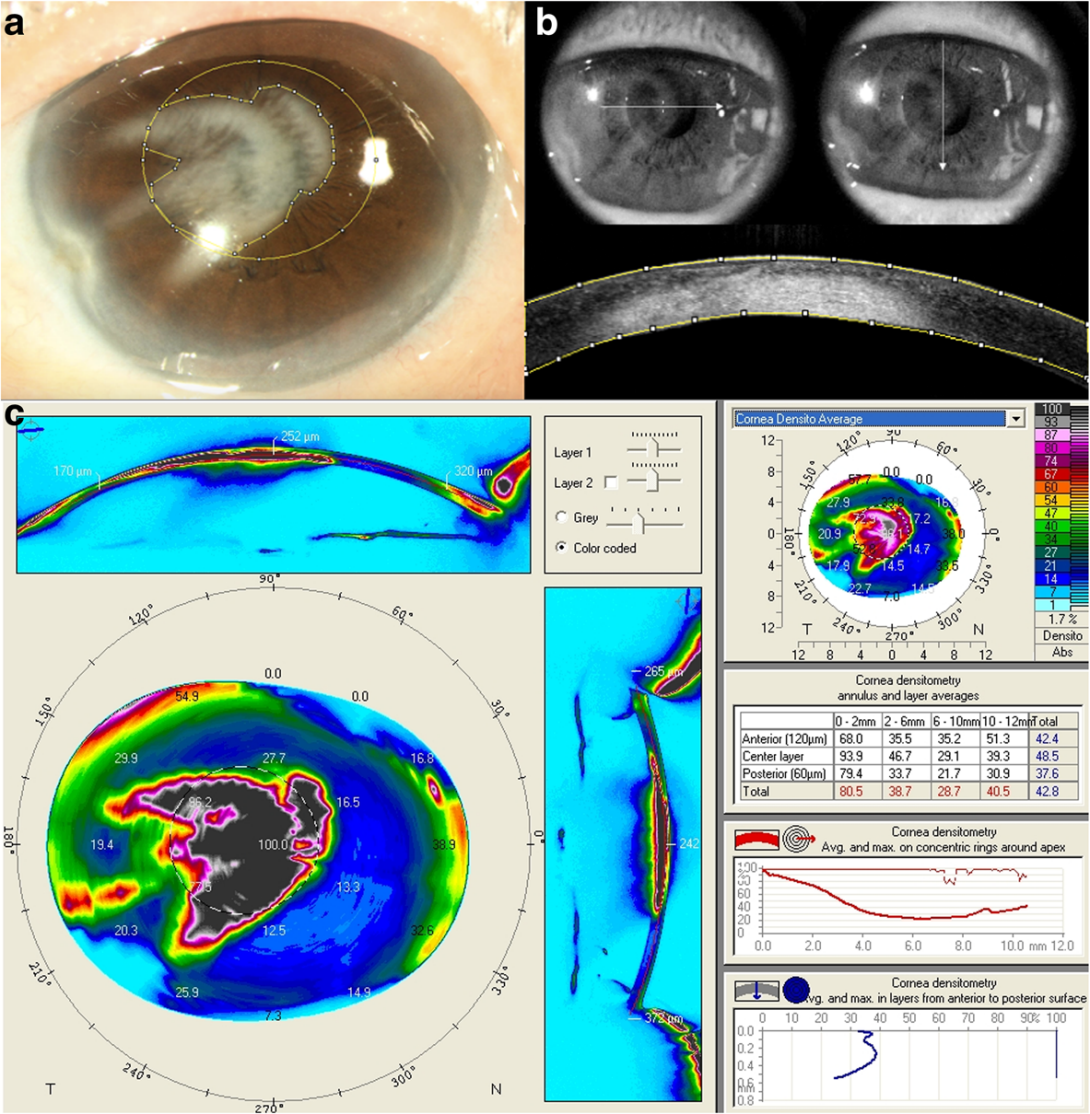
Determination of the severity of corneal opacity by anterior segment OCT. **a** A pupillary-centered ellipse (50% corneal horizontal diameter as the major axis and 50% vertical diameter as the minor axis) was drawn, and the percentage of opacity occupying the central cornea was calculated. **b** To estimate the density of opacity involving the central cornea, OCT images with respect to central horizontal and vertical meridians (arrows in the upper figure) were taken to calculate the reflective signals of the cross-sectional images (lower figure). Corneal opacity density was defined as the mean value of the vertical and horizontal cross sectional signals. Corneal opacity severity = percentage of opacity in the central ellipse cornea x the density of opacity. **c** Standard output of Scheimpflug corneal densitometry of the same eye; the color coded topography map demonstrated the high density of opaque area, which is not irregular and non-homogenous; the right middle table provided the gray scale units including the central 2 mm and 2-6 mm annulus, which is area most relevant to visual performance

The boundary of corneal opacification was demarcated manually (Fig. 1a), and its size was measured using Image J software (National Institutes of Health, Maryland). The percentage of opacity occupying central corneal region was calculated. The central cornea was defined as a central ellipse with 50% corneal horizontal diameter as major axis and 50% vertical diameter as minor axis (Fig. 1a). To quantify the density of the opaque area, vertical and horizontal midline cross-sections were measured by anterior segment OCT (RT-100, Optovue, CA) and analyzed by Image J software (Fig. 1b). Detected signals were given a value ranging from 0 (plain black) to 255 (plain white) according to their reflectivity (Fig. 1b). The above value represented mean gray value of OCT (shown in below formula). Assumption was made that opacity density can be represented by average of mean gray value of vertical and horizontal cross sections. The objective severity of corneal opacification was calculated as the size multiplied by the density as followed:

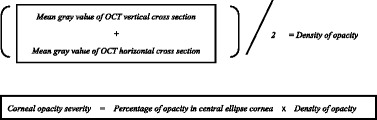



### Corneal densitometry

To measure the backscatter of the cornea, cornea densitometry was analyzed by rotating Scheimpflug camera (Pentacam AXL; Oculus, Wetzlar, Germany). This modality can demonstrate opaque area of entire cornea and express in color-scale units. And the scale is further calibrated by built-in software, which defines minimum light scatter of 0 (most transparent) and maximum light scatter of 100 (total opaque). Due to low repeatability and age-related change effect measurement accuracy outside 6 mm-zone. We only analyzed area within 6 mm central zone diameter measured from corneal apex, and thickness of total corneal layer. Figure 1c demonstrated the result that Scheimpflug Pentacam analyzed corneal densitometry of corneal opacity shown in Fig. 1a.

### Surgical technique

All surgeries were performed under topical anesthesia with same techniques. In brief, a side port was made, and a clear corneal tunnel was fashioned at the 11 o’clock. The viscoelastic device was injected into the anterior chamber, and continuous curvilinear capsulorhexis was performed without dye staining. Phacoemulsification was accomplished using a “divide and conquer” maneuver. Automated irrigation and aspiration were used to remove residual cortex. To calculate IOL power, a preoperative keratometry reading was taken in the ipsilateral eye or from the other eye. Emmetropic IOL power was calculated using the Sanders, Retzlaff and Kraff-T formula. A foldable acrylic lens was implanted. Stromal hydration was performed to seal the wounds. Topical gentamicin was applied to the ocular surface after surgery.

### Postoperative follow-up

The patients were examined on postoperative days 1, 3 and 7, and then 1 month and every 3 to 4 months thereafter. During each follow-up visit, a complete ophthalmic examination including UCVA, BCVA, keratometry, tonometry, slit-lamp examination, endothelial cell density, central corneal thickness and fundus examination was performed.

### Statistical analysis

All data was analyzed by commercially available SPSS statistical software (IBM Corp. Version 20.0, Armonk, NY). A *P* value of less than 0.05 was considered to be statistically significant.

The normality of all data samples was first checked by the Kolmogorov-Smirnov test. Because the data did not fulfill the criteria for normal distribution, the Spearman correlation coefficient was calculated to assess the relationships of clinical corneal haze score, corneal densitometry, and objective OCT corneal opacity grading with best corrected visual acuity. Linear regression was used to produce the line passing through the data.

## Results

### Preoperative and postoperative data

Table 1 summarizes the preoperative demographic features of the patients and characteristics of corneal pathologies. The study consisted of 23 eyes in 19 patients. The mean age at the time of surgery was 72.22 ± 10.1 years old. The most common cause of corneal opacity was infectious keratitis, followed by idiopathic keratopathy and bullous keratopathy. Among them, eleven eyes had posterior segment comorbidities. No perioperative complications were noted. Furthermore, none of the eyes shifted from phacoemulsification to extracapsular cataract extraction intraoperatively, and no intraoperative increase in the intensity of corneal haze was found.

**Table 1:** Demographics and corneal opacity severity of the patients

Case No.	Gender	Laterality	Etiology of corneal opacity	Location of corneal opacity	Percentage of opacity area involving central ellipse (%)	Average density of corneal opacity	Corneal opacity severity*	Associated ocular pathology	Cataract grading
1	F	OS	Traumatic scar	Superotemporal scar involving central cornea	NA	NA	NA		NS3+ CO2+
2	M	OD	Bullous keratopathy	Diffusive opacity sparing temporal side	68.9	70.9	4882	PPA	NS2+
	M	OS	Bullous keratopathty	Inferior focal scar involving central cornea	79.6	67.7	5389	PPA	NS2+ PSCO1+
3	M	OD	Herpetic keratitis	Central scar	41.4	87.1	3602	CR degeneration	NS3+
	M	OS	Herpetic keratitis	Paracentral scar	53.1	73.1	3877	CR degeneration	NS3+
4	M	OS	Herpetic keratitis	Central scar	62.7	101.2	6340		NS2+
5	M	OD	Herpetic keratitis	Central scar	31.1	43.9	1365		NS4+ PSCO3+
6	M	OD	OCP	Diffusive faint scar	NA	NA	NA		NS2+ CO2+ PSCO1+
	M	OS	OCP	Diffusive faint scar	NA	NA	NA		NS2+ CO2+ PSCO1+
7	M	OS	Traumatic scar	Diffusive dense scar	66.5	93.9	6246	CR degeneration	NS1+ CO1+ PSCO1+
8	F	OD	Idiopathic	Central focal scar	59.1	94.7	5592		NS2+
9	F	OS	Herpetic keratitis	Paracentral focal scar	40.0	119	4757	Macular degeneration	NS4+
10	M	OS	Traumatic scar	Central focal scar	NA	NA	NA		Traumatic cataract
11	F	OS	Idiopathic	Paracentral focal scar	NA	NA	NA		NS2+ CO2+
12	M	OS	Idiopathic	Paracentral dense scar	80	97.2	7780	Ambylopia	NS1+
13	F	OD	Herpetic keratitis	Central focal scar	93.8	89	8344		NS2+ CO1+
14	F	OD	SJS	Central scar	14.5	101.3	1470		NS4+
15	F	OS	Idiopathic	Paracentral scar	NA	NA	NA	Ambylopia	Total opacity
16	F	OD	Idiopathic	Diffusive scar	NA	NA	NA	CR degeneration	NS2+ CO2+
	F	OS	Herpetic keratitis	Diffusive scar	NA	NA	NA	CR degeneration	NS2+ CO2+
17	F	OS	Herpetic keratitis	Central scar	76.1	86	6548		NS3+ CO2+
18	F	OD	Neurotrophic keratitis	Diffusive faint scar	30.2	40	1207	Pale disc	NS4+ CO3+
19	M	OD	Fungal ulcer	Central focal scar	NA	NA	NA		NS2+ CO2+

*M* male, *F* female, *OD* right eye, *OS* left eye, *OCP* ocular cicatricial pemphigoid, *SJS* Stevens Johnson syndrome, *PPA* peripapillary atrophy, *CR degeneration* chorioretinal degeneration, *NS* nuclear sclerosis, *CO* cortical opacity, *PSCO* posterior subcapsular opacity, *NA* not accessible

*area percentage of central ellipse x average density

### Refractive and surgical outcomes

**Fig. 2:**
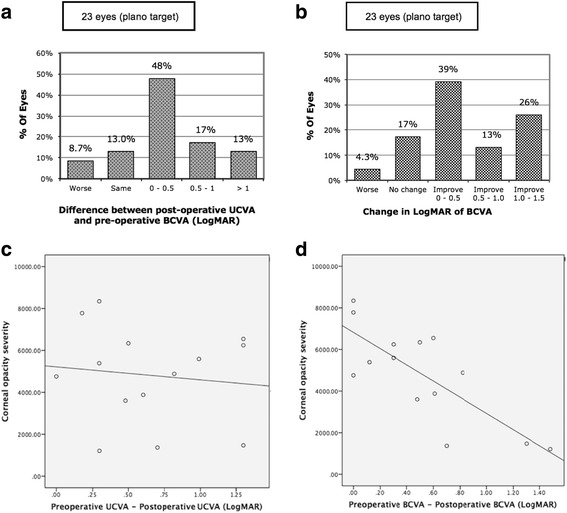
Visual outcomes and the correlation with severity of corneal opacity after cataract surgery in eyes with visually debilitating cataracts and coexisting corneal opacities. **a** Comparison of pre-operative BCVA and post-operative UCVA in logMAR, histogram representing efficacy of cataract surgeries. **b** Comparison of pre-operative BCVA and post-operative BCVA in logMAR, histogram demonstrating safety of surgeries in our cases; only one case (4.3%) become worse in visual performance. **c** There was a statistically significantly negative linear relationship and strong correlation in terms of BCVA (*r* = − 0.782, *P* = 0.001); **d** there was a mildly significantly negative linear relationship and weak correlation regarding UCVA (*r* = − 0.118, *P* = 0.688). (BCVA = best corrected visual acuity; UCVA = uncorrected visual acuity)

Table 2 summarizes the postoperative visual outcomes. The mean follow-up period was 18.57 ± 15.42 months. The preoperative mean UCVA and BCVA was 20/800 and 20/630, which significantly improved to 20/200 and 20/160, respectively (*P* < 0.001). All eyes had an improved UCVA except for three, including two with no change in UCVA and one with a decrease in UCVA from 20/285 to 20/2000. Figure 2a represent the true aim of our study, 92.3% cases achieved visual acuity were as good as or better than that preoperatively with correction. The BCVA improved for all eyes except for four in which the BCVA was unchanged, and one, which lost BCVA postoperatively (Fig. 2b).

**Table 2:** Postoperative visual outcomes and complications

Case No.	Laterality	ECD (Pre- / post-op)	Corneal thickness (Pre- / post-op) (μm)	Pre-op UCVA	Post-op UCVA	Pre-op BCVA	Post-op BCVA	Early Complications (≤ 1 month)	Late Complications (> 1 month)	Secondary procedure	Follow-up period (months)
**1**	OS	NA/NA	484/481	20/200	20/100	20/200	20/100			PKP	24
**2**	OD	893/782	484/517	20/320	20/50	20/320	20/50				15
	OS	1318/1052	403/426	20/100	20/50	20/66	20/50				6
**3**	OD	NA/3106	NA/610	20/200	20/66	20/200	20/66				15
	OS	NA/2688	NA/555	20/2000	20/500	20/2000	20/500				14
**4**	OS	NA/NA	NA/601	20/1000	20/320	20/1000	20/320		Recurrent HSV keratitis		15
**5**	OD	NA/962	NA/569	20/2000	20/400	20/2000	20/400				19
**6**	OD	NA/NA	NA/NA	20/2000	20/2000	20/2000	20/2000		OCP progression		27
	OS	NA/NA	NA/NA	20/285	20/2000	20/300	20/2000		OCP progression		26
**7**	OS	2809/2488	596/615	20/2000	20/100	20/200	20/100				54
**8**	OD	NA/NA	418/406	20/500	20/50	20/100	20/50				26
**9**	OS	NA/NA	400/428	20/2000	20/2000	20/2000	20/2000				12
**10**	OS	NA/NA	NA/735	20/2000	20/100	20/2000	20/66			PKP	10
**11**	OS	NA/NA	NA/552	20/200	20/100	20/200	20/100				19
**12**	OS	1727/1664	567/581	20/100	20/66	20/66	20/66				10
**13**	OD	NA/2364	NA/592	20/200	20/100	20/66	20/66	MCE	Recurrent HSV keratitis		65
**14**	OD	NA/NA	NA/600	20/2000	20/100	20/2000	20/100	Diffusive SPK			13
**15**	OS	2008/495	593/622	20/20000	20/1000	20/20000	20/800				2
**16**	OD	NA/1969	NA/519	20/2000	20/200	20/2000	20/200				3
	OS	NA/NA	NA/412	20/1000	20/400	20/1000	20/400				2
**17**	OS	1555/2127	NA/471	20/2000	20/100	20/400	20/100	MCE	Recurrent HSV keratitis		12
**18**	OD	NA/NA	NA/453	20/2000	20/1000	20/2000	20/66				38
**19**	OD	1007/NA	533/529	20/500	20/100	20/200	20/66				6

*OD* right eye, *OS* left eye, *NA* not accessible, *ECD* corneal endothelial cell density, *OCP* ocular cicatricial pemphigoid, *SPK* superficial punctate keratitis, *HSV* herpetic simplex keratitis, *MCE* microcystic edema, CF counting fingers, *HM* hand motion, *UCVA* uncorrected visual acuity, *BCVA* best corrected visual acuity, *Pre-op* preoperative, *Post-op* postoperative, *PKP* penetrating keratoplasty

**Fig. 3:**
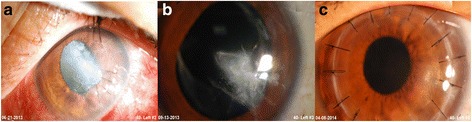
Two stage surgery in case 10 suffering from traumatic corneal perforation and cataract. **a** Initial ocular penetrating trauma with coexisting traumatic cataract, BCVA was counting finger at 10 cm after primary eyeball repair. **b** BCVA achieved 20/66 after cataract surgery. **c** Penetrating keratoplasty was performed 6 months later. BCVA further improved to 20/22. (BCVA = best corrected visual acuity)

The causes of limited visual improvements could be either pre-existing posterior segment disorders such as macular degeneration and amblyopia, or exacerbation of OCP and reactivation of herpetic keratitis after surgery. PKP was performed 6 months after cataract surgery in 2 cases. Both corneal grafts remained clear at the last visit, with improvements in BCVA from 20/100 to 20/22 and from 20/66 to 20/22, respectively (Fig. 3).

### Corneal condition after phacoemulsification

To investigate whether cataract surgery induced corneal decompensation, changes in corneal endothelial count were analyzed. The mean preoperative corneal endothelial cell density (1718.33 ± 267.19 cells/mm^2^) was not significantly different from that after surgery (1434.67 ± 321.29 cells/mm^2^ (*p* = 0.242) with a mean endothelial loss of 283.66 ± 681.71 cells/mm^2^. The percentage of endothelial loss was 14%, which is similar to that reported (8 to 14%) after phacoemulsification in normal corneas [22, 23]. However, due to poor corneal transparency, the preoperative endothelial cell density was only accessible in seven eyes and eleven eyes postoperatively. Therefore, we used changes in corneal thickness as a surrogate for corneal endothelium dysfunction [16]. Complete corneal thickness data were obtained in nine eyes, and the mean preoperative corneal thickness was 497.56 ± 79.08 um and 509.22 ± 79.06 um postoperatively. There was no statistically significant perioperative difference (*P* = 0.055), indicating that the endothelium was not compromised after surgery.

### Association of the severity of corneal opacity and surgical outcomes

**Fig. 4:**
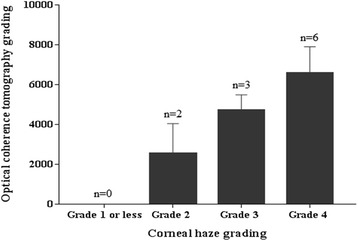
Corneal haze grading related to grading with optical coherence tomography. Error bar = standard error of mean; *n* = observation number

**Fig. 5:**
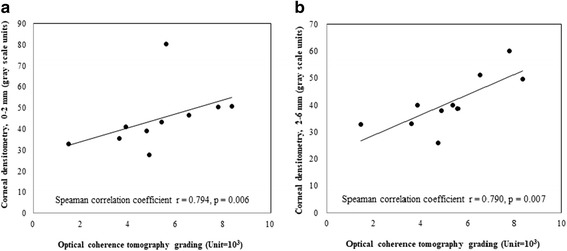
Scatterplot shows a significant correlation between optical coherence tomography grading with corneal densitometry in central (**a**) the central 2 mm (Spearman correlation *r* = 0.794, *P* = .006), (**b**) 2-6 mm annulus. (Spearman correlation *r* = 0.79, *P* = .007)

**Fig. 6:**
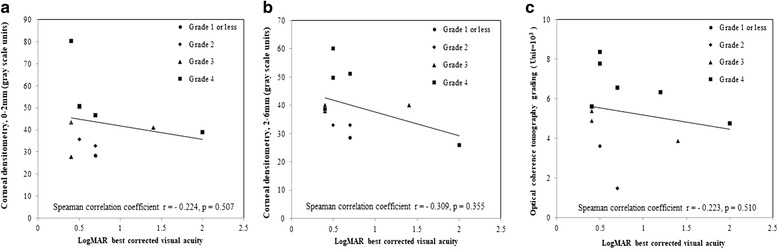
Scatterplot showing no significant correlation between logMAR best corrected visual acuity and corneal densitometry (**a**) central 2 mm (Spearman correlation *r* = − 0.224, *P* = .507) (**b**) 2–6 mm annulus ring (Spearman correlation *r* = − 0.309, *P* = .355) or (**c**) optical coherence tomography grading (Spearman correlation *r* = − 0.223, *P* = .510)

Figure 4 demonstrates corneal haze grading increase with their OCT grading for opacity severity. Also, we found significant correlation of OCT grading with corneal densitometry within central 2 mm and 2 to 6 mm diameter (Spearman correlation *r* = 0.794, *P* = .006 and *r* = 0.790, *P* = .007 respectively) (Fig. 5). Regarding visual outcome, there is no significant correlation between logMAR BCVA and corneal densitometry (2 mm, *r* = − 0.224, *P* = .507; 2 to 6 mm, *r* = − 0.309, *P* = .355) and also OCT grading (*r* = − 0.223, *P* = .510) (Fig. 6). We performed Pearson correlation analysis to investigate the association between postoperative visual outcomes improvement and the OCT grading method. Among the 14 eyes available for anterior segment OCT examinations, there was only a weak but insignificant correlation with improvements in UCVA postoperatively (*r* = − 0.118, *P* = 0.688, Fig. 2c); in the other hand, the opacity grading value was negatively correlated with postoperative improvements in BCVA (*r* = − 0.78, *P* < 0.05, Fig. 2d). Our results suggest that the more severe the corneal opacity, the lesser the improvements in BCVA after cataract surgery.

## Discussion

The presence of corneal opacities can be challenging for surgeons during cataract surgery and combined corneal transplantation may be preferred. However, patients may hesitate to receive PKP for only mild-to-moderate nebulae. For one-eyed patients whose corneal opacities are accompanied by deep stromal vascularization, it may not be appropriate to perform corneal transplantation due to high-risk of graft rejection [8]. Therefore, cataract surgery may be an alternative in these patients [12].

Panda et al. reported that phacoemulsification with IOL implantation with 0.06% trypan blue improved BCVA from less than 40/200 to 20/60 [12]. A similar study reported that 64.7% of patients improved visual acuity, and that 47.1% of those with advanced cataracts achieved 20/100 or better [11]. In this study, 78% improved BCVA, with 60.8% achieving better than 20/100 postoperatively. Although final visual acuity was suboptimal, the patients had improvements of at least two lines except for one eye with worse BCVA. Cataract surgery alone can also be an interim procedure for patients awaiting donor corneas. Two cases suffered from corneal lacerations and traumatic cataracts had improved vision after cataract surgery with a final BCVA of 20/22 after corneal transplantation (Fig. 3).

Corneal haze impedes visibility even with adequate fundus glow. Various techniques were used to improve visualization through opaque corneas during surgery [24]. To optimize visualization, attempts were made to adjust light setting and the following maneuvers resulted in high success rate. First, avoid the corneal opaque area and look for a transparent window to initiate continuous curvilinear capsulorhexis. Visualization during phacoemulsification in eyes with corneal opacity superimposed with mature cataracts can be extremely difficult and technically demanding, therefore we suggest that this procedure be reserved for experienced surgeons. Second, obtain favorable contrast in the surgical field by adjusting the illumination to low or medium intensity, as coaxial lighting of most operating microscopes may interfere with the surgical field due to backscatter and reflection of light from the cornea [25]. Finally, perform “continuous” capsulorhexis with a constant tethered force under the opaque area. Non-continuous capsulorhexis is likely to cause radial tearing and intraoperative complications are even harder to manage in eyes with poor corneal clarity. Therefore, patients must be aware of alternative surgical options and secondary surgeries during preoperative consultation.

Ocular surface diseases such as OCP and viral keratitis can be exacerbated by uneventful cataract surgery and limit surgical outcomes. Preoperative recognition and effective management can prevent devastating complications [26]. The safety and efficacy of cataract surgery has been reported in patients with OCP; however, reactivation of OCP is possible and results in poor outcomes [27]. In a previous study, postoperative visual acuity was worse in eight of 15 eyes by about 2 years due to progressive cicatricial disease [28]. Similar to their findings, case 6 with OCP had a temporary visual improvement in early postoperative period, but visual acuity deteriorated 2 years later because of missed follow-up and uncontrolled disease. In contrast, Puranik et al. argued that surgeries were not associated with exacerbations of inflammation, and that stable visual outcomes could be anticipated in spite of ongoing disease [29]. However, we strongly recommend adequate preoperative immunosuppression therapy and the use of small corneal incisions in cases with OCP scheduled for cataract surgery in order to ensure better outcomes.

The onset of new and recurrent herpetic simplex virus (HSV) keratitis has been reported after uncomplicated cataract surgery [24]. Triggers for HSV keratitis such as surgical trauma and use of steroids have been proposed. Barequet et al. reported that new HSV keratitis may develop after uneventful surgery within 1–5 months under corticosteroid treatment [30]. In this series, three eyes with preexisting herpetic keratitis had signs of reactivation with a mean inactive period of 14.8 weeks. All patients were under topical steroids for at least 4 weeks, and the mean flare-up time of HSV keratitis was 10 months. All eyes were treated with oral acyclovir or topical acyclovir ointment, and the lesions resolved over a mean period of 4 weeks. Unfortunately, two eyes developed further recurrence, and the final visual outcomes were not favorable due to exacerbation of HSV keratitis. Although herpetic eye disease study suggested that prophylactic oral antivirals for 1 year could be effective in preventing half of ocular and non-ocular cases of recurrence in patients with previous HSV keratitis (Herpetic Eye Disease Study Group 1998), no guidelines for antiviral prophylaxis currently exist for cataract surgery [26]. To prevent HSV exacerbations compromising well-tolerated cataract surgery, we suggest that oral antiviral prophylaxis in cases of suspected reactivation of HSV keratitis.

Recently, OCT has been shown a valuable tool to detect and analyze corneal opacities in different clinical situations [13, 31]. Accumulating evidence has demonstrated that cross-sectional images of the cornea in anterior segment OCT correlate well with the morphology in light microscopy [13]. Wirbelauer and Pham (2004) reported the quantitative assessment of calcified corneal lesions using anterior segment OCT. They found that reflectivity in OCT could be used to represent the severity of opacities, and concluded that OCT was helpful in quantifying the corneal opacity and in guiding the treatment plan. However, the parameters in their study included only depth of the calcified lesions and thickness of the corneas [16]. Normative corneal densitometry in healthy subjects using Oculus Pentacam system and its relation with refractive parameter were published before [32]. Effect of backscatters from corneal pathology on visual performance had also been proven by Kamiya et al. [17, 18]. However, no quantitative analysis using ASOCT and its relations with corneal densitometry and visual outcome has been so far elucidated.

Current study took into consideration the area and density of the corneal opacity involving the pupillary axis, and found that the more severe the opacity index according to the reflectivity signal, the less likely BCVA improvements would be achieved after surgery (Fig. 2d). The similar interfering effect of backward scattering has also been studied but no definite association was proved with corneal densitometry [17, 18]. This is might due to the similar conditions that we found in our study, Pentacam performed poorly to analyzed corneal opacity in moderate-to-high density, location closed to pupil center and pathology concentrated at superficial cornea. Also, the circle or annuli area Pentacam system analyzed is not useful if the opacity area is irregular in shape. In contrasts, one can delineate along contour of opacity area and quantified with the depth detected by ASOCT.

Confounding parameters such as the thickness of the cornea, irregularities of the corneal surface and refractive index in the opacified area were controlled for by comparing preoperative and postoperative visual acuity in the same individual. Because of the simplicity and reproducibility of OCT measurements, surgeons may adopt our proposed grading system as a useful guide to predict visual outcomes in patients who are not candidates or hesitate for combined PKP and cataract surgery.

Although anterior segment OCT allows for non-invasive and objective analysis of corneal structural abnormalities and has good predictability for postoperative BCVA, some limitations still exist. For example, simply averaging vertical and horizontal cross-sectional lines is inadequate to represent the size and shape of corneal opacities due to their non-homogenous properties. Severity of cataract also contributes to the improvement of visual acuity. Above two factors may be reason why we did not find an association between corneal opacity severity and improvements in UCVA. Furthermore, corneal surface irregularities and tearing that may contribute to visual disturbance were not addressed. Finally, due to the retrospective nature, the effects of underlying posterior segment pathologies on visual outcomes were not excluded. Therefore, future prospective studies with well-designed anterior segment OCT software and strict criteria to exclude the confounding factors are warranted to investigate the effects of corneal opacity on visual outcomes after cataract surgery.

## Conclusions

In conclusion, phacoemulsification is not an alternative to PKP, however it is a safe and feasible method for patients who are poor candidates for transplantation, especially for those with vision in only one eye and who are unable to comply with follow-up protocols after keratoplasty. Moreover, phacoemulsification can also serve as an interim procedure to allow patients to become ambulatory while they are waiting for PKP, especially in developing countries with a lack of good quality donor corneas.

## Abbreviations

BCVA: Best corrected visual acuity
CF: Counting finger
HSV: Herpetic simplex virus
IOL: Intraocular lens
OCP: Ocular cicatricial pemphigoid
OCT: Optical coherence tomography
PKP: Penetrating keratoplasty
UCVA: Uncorrected visual acuity
